# An overview protocol of biomarkers for breast cancer detection

**DOI:** 10.1097/MD.0000000000016024

**Published:** 2019-06-14

**Authors:** Zhijuan Sheng, Jing Wang, Muyang Li, Xinyue Luo, Runjin Cai, Mei Zhang

**Affiliations:** a Department of Galactophore, Gansu Provincial Cancer Hospital; b Department of Obstetrics and Gynecology, First Hospital of Lanzhou University; c The Second Clinical Medical College of Lanzhou University; d Department of Radiology, Gansu Provincial Cancer Hospital, Lanzhou, China.

**Keywords:** adjusted indirect comparison, biomarker, breast cancer, diagnostic test accuracy, overview

## Abstract

**Background:**

Among females, breast cancer is the most commonly diagnosed cancer and the leading cause of cancer death over 100 countries. Generally, the prognosis of early-stage breast cancer is good. However, the prognosis is very poor when the disease is diagnosed at an advanced stage. Many screening methods have been used for early detection of breast cancer, but there are some limitations of these methods. Recently, some systematic reviews have evaluated the value of biomarkers for detecting breast cancer. However, most of the systematic reviews (SRs) only evaluated the diagnostic value of 1 biomarker, and it is unclear which biomarker is the best diagnostic test for breast cancer. This overview aims to assess the methodological and reporting quality of available systematic reviews and to compare the diagnostic value of different biomarkers.

**Methods:**

PubMed, Embase.com, the Cochrane Library of Systematic Reviews, and Web of Science were searched to identify published systematic reviews reporting the value of biomarkers for detecting breast cancer. Title and abstracts, as well as full texts, were screened in duplicate based on inclusion and exclusion criteria. The Assessment of Multiple Systematic Reviews-2 (AMSTAR-2) tool and Preferred Reporting Items for Systematic Reviews and Meta-analysis diagnostic test accuracy (PRISMA-DTA) checklist will be used to assess the methodological and reporting quality, respectively. We will conduct the pairwise meta-analysis and indirect comparisons using STATA 13.0.

**Results:**

The results of this study will be published in a peer-reviewed journal

**Conclusion:**

This overview will provide comprehensive evidence of different biomarkers for the diagnosis of breast cancer.

**PROSPERO registration number:**

CRD42019125880.

## 1 Introduction

Among females, breast cancer is the most commonly diagnosed cancer, with 2.1 million newly diagnosed cases in 2018, accounting for almost 25% cancer cases among women.^[1]^ Breast cancer is also the leading cause of cancer death over 100 countries, contributing to 15% of all cancer deaths in 2018.^[1]^ In the past few decades, the incidence of breast cancer in most transition countries has been rising, especially in Asia, South America, and Africa.^[1,2]^ Generally, the prognosis of early-stage breast cancer is good and the overall 5-year survival rate is higher than 90%.^[3,4]^ However, the prognosis of advanced breast cancer is poor and the overall 5-year survival rate is only about 20% if diagnosed at a late stage when the malignant cells have already spread to other organs.^[3–6]^ Therefore, early detection or screening for breast cancer is important to improve the general prognosis of this disease.^[7]^

Many screening methods have been used for early detection of breast cancer, such as breast self-examination, mammography, ultrasonography, mammography, and exfoliative cytology.^[8,9]^ Although these techniques increase the detection rate of early breast cancer, there are still some limitations.^[9]^ Therefore, enormous efforts have been exerted to explore biomarkers for the early diagnosis of breast cancer. Scholars have conducted some systematic reviews (SRs) to assess the diagnostic value of microRNA, prostate-specific antigen, and circulating cell-free DNA for detecting breast cancer and some biomarkers showed potential diagnostic value.^[10–12]^ As we know, well-conducted SRs and meta-analyses with high quality can provide the best evidence for clinical practice and healthcare decisions.^[13–15]^ However, the methodological and reporting quality of these SRs remains unclear. What is more, most of the SRs evaluated the diagnostic value of only 1 biomarker, and it is also not clear which biomarker has superior performance for early detection of breast cancer. Thus, this overview aims to assess the diagnostic accuracy of biomarkers for breast cancer, explore the methodological and reporting quality of available SRs, and to compare the diagnostic accuracy of different biomarkers with adjusted indirect comparisons.

## 2 Methods

This overview of systematic reviews will be reported according to the Preferred Reporting Items for Systematic Reviews and Meta-analysis^[16]^ checklist. As a part of our project, this protocol has been registered on international prospective register of systematic review (PROSPERO) (CRD42019125880).

### 2.1 Study inclusion and exclusion criteria

#### 2.1.1 Inclusion criteria

The studies fulfilling all the following criteria will be included in the systematic review.

- Participants: All patients diagnosed with breast cancer were confirmed by pathological examination will be included, regardless of their age, race, or nationality. There are no restrictions on the treatment plan, tumor stage, and pathological type.
- Interventions: Any biomarker used for the diagnosis of breast cancer, such as microRNAs and carcinoembryonic antigen. We will also include intervention of combined biomarkers but 1 biomarker combines with imaging modalities or other index tests will be excluded.
- Outcomes: Diagnostic performance indices of biomarkers for breast cancer including sensitivity, specificity, positive likelihood ratio, negative likelihood ratio, positive predictive value, negative predictive value, diagnostic odds ratio, and area under the curve.
- Study design: Systematic reviews evaluating the value of biomarkers for diagnosing breast cancer. The included studies of the systematic reviews can be randomized controlled trials, cohort studies, case-control studies, or cross-sectional studies. The SRs should include clear sensitivity, specificity, inclusion/exclusion criteria, and adequate search strategy.

### 2.2 Exclusion criteria

- SRs that reported diagnostic value of imaging modalities.
- Studies did not have a full text or any conference abstracts.
- Review articles; guidelines, consensus, documents, or expert position papers; comments, letters, brief reports, proceedings, or protocol studies.

### 2.3 Search strategy

Experienced medical information experts worked with the review team to develop a comprehensive search strategy.^[17]^ The relevant search terms were translated into the appropriate vocabulary and grammar for the databases. We searched the following databases from their inception to February 2019: the Cochrane Library of Systematic Reviews, PubMed, Web of Science, and Embase.com. The search was not restricted by language or publication status. We have also searched the reference lists of relevant SRs for potential eligible studies. The detailed search strategy of the Cochrane library is presented as following:

1. MeSH descriptor: [Breast Neoplasms] explode all trees
2. MeSH descriptor: [Breast Carcinoma In Situ] explode all trees
3. MeSH descriptor: [Breast Neoplasms, Male] explode all trees
4. MeSH descriptor: [Carcinoma, Ductal, Breast] explode all trees
5. MeSH descriptor: [Carcinoma, Lobular] explode all trees
6. MeSH descriptor: [Inflammatory Breast Neoplasms] explode all trees
7. MeSH descriptor: [Triple Negative Breast Neoplasms] explode all trees
8. MeSH descriptor: [Unilateral Breast Neoplasms] explode all trees
9. breast neoplasm∗:ti,ab,kw OR breast tumor∗:ti,ab,kw OR breast carcinoma∗:ti,ab,kw OR breast cancer∗:ti,ab,kw OR breast tumour∗:ti,ab,kw OR mammary neoplasm∗:ti,ab,kw OR mammary tumor∗:ti,ab,kw OR mammary carcinoma∗:ti,ab,kw OR mammary cancer∗:ti,ab,kw OR mammary tumour∗:ti,ab,kw OR breast adenocarcinoma∗:ti,ab,kw OR breast carcinogenesi:ti,ab,kw OR breast sarcoma∗:ti,ab,kw OR phyllodes tumor∗:ti,ab,kw OR intraductal carcinoma∗:ti,ab,kw OR lobular carcinoma∗:ti,ab,kw
10. #1 OR #2 OR #3 OR #4 OR #5 OR #6 OR #7 OR #8 OR #9
11. MeSH descriptor: [Sensitivity and Specificity] explode all trees
12. MeSH descriptor: [False Positive Reactions] explode all trees
13. MeSH descriptor: [False Negative Reactions] explode all trees
14. MeSH descriptor: [ROC Curve] explode all trees
15. MeSH descriptor: [Predictive Value of Tests] explode all trees
16. sensitivity:ti,ab,kw OR specificity:ti,ab,kw OR “receiver operating characteristic”:ti,ab,kw OR “receiver operator characteristic”:ti,ab,kw OR “predictive value∗”:ti,ab,kw OR roc:ti,ab,kw OR “pre-test odds”:ti,ab,kw OR “pretest odds”:ti,ab,kw OR “pre-test probabilit∗”:ti,ab,kw OR “pretest probabilit∗”:ti,ab,kw OR “post-test odds”:ti,ab,kw OR “posttest odds”:ti,ab,kw OR “post test probabilit∗”:ti,ab,kw OR “posttest probabilit∗”:ti,ab,kw OR “likelihood ratio∗”:ti,ab,kw OR “positive predictive value∗”:ti,ab,kw OR “negative predictive value∗”:ti,ab,kw OR “false negative∗”:ti,ab,kw OR “false positive∗”:ti,ab,kw OR “true negative∗”:ti,ab,kw OR “true positive∗”:ti,ab,kw
17. #11 OR #12 OR #13 OR #14 OR #15 OR #16
18. #10 AND #17

### 2.4 Study selection

We imported the retrieved records into EndNote X8 (Thomson Reuters (Scientific) LLC Philadelphia, PA) to remove redundancies. Then, 2 independent reviewers read the titles and abstracts of the identified studies; the records that do not meet the inclusion criteria were deleted. The full text of all possibly relevant studies was screened and assessed by the same 2 reviewers to determine if they meet the eligibility criteria. Two researchers checked whether each study is suitable for our analysis. Detailed reasons for eliminating studies were documented. The discrepancies that arise were dealt with by deliberation and debate between the 2 authors. If an agreement cannot be reached, a third reviewer was consulted.

### 2.5 Data extraction and management

Data will be extracted from the systematic reviews by 2 independent reviewers. We will develop a standardized, Microsoft Excel master sheet framework to record data extracted from each SR. The extracted data will include the following items: author, country of corresponding author, number of authors, publication year, journal name, country of journal, funding, disease, number and name of biomarkers, number and name of reference test, types of included studies, number of included studies, samples, pooled sensitivity, specificity, likelihood ratio, diagnostic odds ratio, area under curve, and their 95% confidence interval (CI). If the diagnostic performance indices did not report in the SRs, we will use the data of true positive, false positive, true negative, false negative values to calculate the pooled sensitivity, specificity, and diagnostic odds ratio. If we identify multiple reviews addressing the same research question but share the same primary study, we will include the most recent review and the duplicated SRs will be used as a supplement to the data. Disagreements will be resolved by consensus or by discussion with a third reviewer.

### 2.6 Assessment of methodological and reporting quality

The methodological quality of the included SRs will be assessed using the Assessment of Multiple Systematic Reviews-2 (AMSTAR-2) tool, which can be used to evaluate the quality of SRs based on non-RCTs.^[18,19]^ The AMSTAR-2 tool is a revised revision of the original AMSTAR instrument, which is a reliable methodological quality that has a good agreement, construct validity and feasibility.^[20–22]^ It contains 16 items, among which 7 are critical domains. The overall confidence of the results of the review will be classified as high, moderate, low, and critically low, and each item will be responded to “Yes”, “No”, or “Partial Yes”. The reporting quality of included SRs will be assessed according to the Preferred Reporting Items for Systematic Reviews and Meta-analysis diagnostic test accuracy (PRISMA-DTA) checklist.^[23]^ To indicate the degree of compliance, each checklist item will be assigned one of the following 3 responses: “Yes” for total compliance; “Partial” for partial compliance; and “No” for non-compliance.^[24]^ Two of the evaluators will independently assess the quality of all the included SRs, and any disagreement between the evaluators will be resolved by discussion or consultation with a third evaluator.

### 2.7 Dealing with missing data

If there is no specific or insufficient data in the published SRs, the author will be contacted by email, or telephone to provide the necessary information. The data will be discarded if we fail to get sufficient data. The analysis will be conducted based on available data, and the potential impact of missing data will be discussed.

### 2.8 Measures of treatment effect

To summarize the diagnostic value of each biomarker, pooled sensitivity, specificity, diagnostic odds ratio (DOR), positive likelihood ratio, negative likelihood ratio, and their 95% CI will be used.

### 2.9 Assessment of heterogeneity

The heterogeneity of the study results will be analyzed by chi-squared test and determined using the I^2^ value. If the I^2^ is less than 50%, the statistical heterogeneity between tests can be ignored, and the effect size will be estimated using a fixed-effect model. If I^2^ exceeds 50%, there is considerable heterogeneity between tests; we will explore sources of heterogeneity by subgroup analysis and meta-regression.

### 2.10 Data synthesis

#### 2.10.1 Pairwise meta-analysis

STATA (13.0; Stata Corporation, College Station, TX) software will be used to perform pairwise meta-analysis with the data of pooled sensitivity, specificity, DOR, positive likelihood ratio, negative likelihood ratio and their 95% CI lower limit, 95% CI upper limit extracted from each SR. If there is no statistical heterogeneity between the SRs, a fixed-effect model will be used for the meta-analysis. If there is statistical heterogeneity, we will analyze the sources of heterogeneity. If there is no clinical heterogeneity, the random effects model will be used to perform the meta-analysis. Otherwise, clinical heterogeneity will be explored through discussion with the review team.

#### 2.10.2 Adjusted indirect comparisons

We will calculate relative diagnostic outcomes between index tests including relative sensitivity, relative specificity, and relative DOR. Then, we will conduct indirect comparisons using relative diagnostic outcomes.

#### 2.10.3 Subgroup analysis and meta-regression

If there is considerable heterogeneity in the included SRs, we will conduct a subgroup analysis or meta-regression based on the types of breast cancer, the country in which the study was conducted, and the age of patients if sufficient data are available.

### 2.11 Assessment of publication bias

To assess the publication bias, the Egger test will be conducted if more than 10 SRs is available for a biomarker.

## 3 Results of study selection

**Figure 1:**
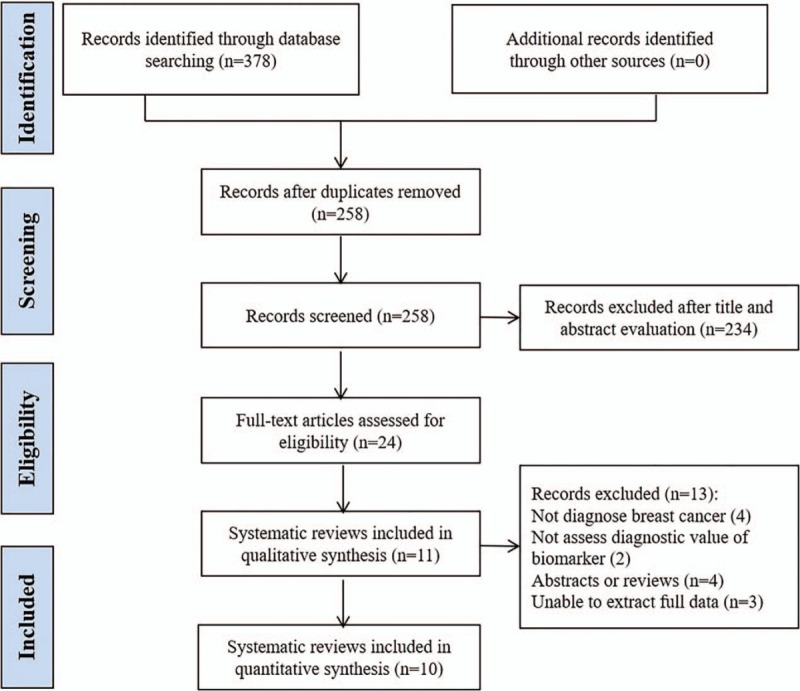
The PRISMA flow chart of literature section. PRISMA = Preferred Reporting Items for Systematic Reviews and Meta-analysis.

The electronic searches identified 378 potentially relevant publications, of which 120 duplicates were removed and 258 records proceeded to title/abstract screening. The remaining 24 SRs were retrieved for full text for further eligibility, and 11 SRs met the a priori criteria and were included. The PRISMA flow chart of literature section is presented in Fig. 1.

## 4 Ethics and dissemination

Ethical approvals and patient consent are not necessary because this overview will be based on published SRs. We will submit our overview to a peer-reviewed journal for publication.

## Author contributions

ZJS, JW, and MZ planed and designed the research. ZJS, JW, MYL, XYL, and RJC tested the feasibility of the study. ZJS and MZ provided methodological advice, polished and revised the manuscript. ZJS, JW, and MZ wrote the manuscript. All authors approved the final version of the manuscript.

**Conceptualization:** Zhijuan Sheng, Jing Wang, Xinyue Luo, Mei Zhang.

**Data curation:** Muyang Li, Xinyue Luo, Runjin Cai.

**Formal analysis:** Zhijuan Sheng, Jing Wang, Mei Zhang.

**Funding acquisition:** Zhijuan Sheng.

**Investigation:** Jing Wang, Muyang Li, Xinyue Luo, Runjin Cai, Mei Zhang.

**Methodology:** Zhijuan Sheng, Jing Wang, Mei Zhang.

**Project administration:** Mei Zhang.

**Resources:** Jing Wang, Muyang Li, Runjin Cai.

**Software:** Zhijuan Sheng, Jing Wang.

**Supervision:** Mei Zhang.

**Validation:** Mei Zhang.

**Visualization:** Zhijuan Sheng, Jing Wang, Muyang Li.

**Writing – original draft:** Zhijuan Sheng, Jing Wang, Mei Zhang.

**Writing – review & editing:** Zhijuan Sheng, Mei Zhang.
